# Molecular genetics and phenotype of 26 Vietnamese patients with congenital hyperinsulinism

**DOI:** 10.1186/1687-9856-2013-S1-P179

**Published:** 2013-10-03

**Authors:** Vu Chi Dung, Nguyen Thanh Liem, Bui Phuong Thao, Nguyen Ngoc Khanh, Can Thi Bich Ngoc, Nguyen Thi Hoan, Khu Thi Khanh Dung, Le To Nhu, Dang Anh Duong, Nguyen Phu Dat, Sarah Flanagan, Sian Ellard

**Affiliations:** 1 Vietnam National Hospital of Pediatrics, Hanoi, Vietnam; 2 Peninsula Medical School, Institute of Biomedical and Clinical Science, Exeter, UK

## 

Hyperinsulinemic hypoglycemia (HH) is a consequence of unregulated insulin secretion by pancreatic β-cells and is a major cause of hypoglycemic brain injury and mental retardation. Congenital HH is caused by mutations in genes involved in regulation of insulin secretion, seven of which have been identified (*ABCC8*, *KCNJ11*, *GLUD1*, *CGK*, *HADH*, *SLC16A1* and *HNF4A*). Severe forms of congenital HH are caused by mutations in *ABCC8* and *KCNJ11*, which encode the two components of the pancreatic β-cell ATP-sensitive potassium channel (sulfonylurea receptor SUR1 and the inwardly rectifying ion channel KIR6.2). Activating mutations in the subunit genes of ATP-sensitive potassium channel can result in monogenic diabetes, whereas inactivating mutations are the most common cause of congenital hyperinsulinism of infancy.

We aim to identify mutations of ABCC8; KCNJ11 and *HNF4A* in Vietnamese patients with congenital HH, to describe the phenotype, and to evaluate outcome of these patients.

This is a case series study including phenotype, genotype characteristics and outcome. Twenty six Vietnamese probands with congenital HH were analyzed for alterations in *ABCC8; KCNJ11* and *HNF4A*. All exons of *KCNJ11; ABCC8* and *HNF4A* genes were amplified from genomic DNA and directly sequenced. In patients with detected mutations, the parental origin of each mutation was determined. 13/26 cases with no identified mutations of *ABCC8; KCNJ11* and *HNF4A* were stable with medical treatment (diazoxide-responsive congenital HH). Eleven probands had mutations in the *ABCC8* and no mutation in the *KCNJ11* and *HNF4A*. Six patients were homozygous or compound heterozygous for the mutations, indicating diffuse pancreatic disease. Their blood glucose levels were normal after nearly total pancreatectomy by laparoscopy. In five patients, heterozygous and paternally inherited mutations were found, suggesting focal disease before surgery. Of which one case had diffuse lesions on histopathology examination and normal blood glucose level after pancreatectomy, one case had diabetes after two days of surgery and stop of insulin injection after 6 months of treatment and one case still needs octreotide treatment for normal blood glucose levels. Altogether, 9 different ABCC8 mutations including three novel alterations (p.F686I, p.I395F and p.G1379S) and six reported mutations (p.F686S, IVS27-1G>A, p.R999X, c.1467+5G>A, p.R934X and p.S1387del) were identified. One case of responsive with diazoxide had partenal inherited mutation in *KCNJ11* [c.482C>T (p.A161V)] and one case of responsive with diazoxide had a novel mutation and maternal inheritance in *HNF4A* [c.659T>C (p.L220P)]. Our results extend the knowledge of the molecular genetics, phenotype and outcome behind congenital HH in Vietnam.

